# Pathogenicity of field strain of fowl aviadenovirus serotype 11 isolated from chickens with inclusion body hepatitis in Morocco

**DOI:** 10.1371/journal.pone.0261284

**Published:** 2021-12-16

**Authors:** Samira Abghour, Mohamed Mouahid, Sami Darkaoui, Jaouad Berrada, Khalil Zro, Faouzi Kichou

**Affiliations:** 1 Division of Pharmacy and Veterinary Inputs, ONSSA, Rabat, Morocco; 2 Hassan 2nd Institute of Agronomy and Veterinary Medicine, Rabat, Morocco; 3 Mouahid’s Veterinary Clinic, Temara, Morocco; 4 Department of Development of Production Sectors, Ministry of Agriculture and Maritime Fisheries, Rabat, Morocco; University of Nicolaus Copernicus in Torun, POLAND

## Abstract

Outbreaks of inclusion body hepatitis have emerged in Morocco since 2013 and has resulted in significant economic losses to poultry farms. Three isolates of the causative virus, Fowl adenonovirus (FAdV)were characterized from chickens with IBH, but their pathogenicity has never been investigated. In this work, the pathogenicity of an isolate FAdV 11 (MOR300315 strain) was evaluated by inoculating a group of 40 SPF chickens at 3 days of age by oral route. A group of 40 chicks injected with phosphate-buffered saline solution was used as a control group. The infected chickens showed decreased weight gain from 3dpi. Necropsy displayed pallor and enlargement in liver, swelling and slight hemorrhage in kidney and spleen at 6 dpi. Histopathological changes were mainly characterized by severe and extensive hepatic necrosis associated with the presence of basophilic intra-nuclear inclusion bodies within hepatocytes. The FAdV was reisolated in chicken embryo fibroblast cell culture from liver tissue homogenate of infected chicken from 3 to 6 dpi. Viral DNA was detected by PCR in liver, kidney, spleen and cloacal swabs from 3 to 13 dpi. Antibody response against inoculated FAdV was appeared from 9 dpi. These results confirmed that the FAdV 11 strain is pathogenic in chicken. This study is the first experimental infection of FAdV 11 in chicken in Morocco, which increase our understanding of its pathogenicity in chickens and indicate that preventive measures against FAdV infection in poultry farms should be implemented in Morocco.

## Introduction

Inclusion body hepatitis (IBH) in chickens, first reported in the United States in 1963 [1], has become one of the major poultry diseases in many countries, including Morocco. Recent reports indicate a sharp rise in severity and occurrence of the disease in Morocco [2–6].

IBH in chickens is characterized by sudden deaths, massive liver necrosis and the presence of intranuclear inclusion bodies in hepatic cells. The disease is commonly noticed in 3 to 7 weeks old broilers and occasional incidence in older broilers and layers [7, 8]. The affected flocks show growth losses and the 2–30% mortalities further aggravate the extent of economic losses [8–10]. Based on the worldwide distribution and ubiquitous presence of adenovirus in healthy poultry flocks, most of the fowl adenovirus are considered to be nonpathogenic [7, 11]. However, some FAdVs including serotype 11, appear to be pathogenic and are associated with clinical manifestations of IBH in chickens [12–15]. Earlier, it was suggested that immune suppression due to preinfection or concurrent infection with infectious bursal disease virus (IBDV) or chicken anemia virus (CAV) induces IBH in chickens [16–19]. However, old and recent studies suggested that IBDV and CAV infections or other immune suppression factors may not be needed for the onset of FAdV based IBH in chickens [9, 20–23].

Pathogenicity of FAdVs varies with the strain or serotype of the virus, age, line and the immune status of the birds [24–28] as well as the route of inoculation in experimental studies. So far, there is no concrete molecular evidence which differentiates FAdVs based on their virulence. Pallister et al. (1996) have differentiated pathogenic FAdV-8 strains from apathogenic strains by using fiber gene sequence [29]. However, it is not applicable to other FAdVs species [27]. The most reliable method to determine the pathogenicity of FAdVs is animal challenge and assessment of mortality and severity of the associated lesions. Several researchers have used a SPF chicken model to study FAdV pathogenicity [24, 30–34].

In Morocco, the virus involved in reported IBH cases have been molecularly identified [5, 6] and the FAdV-11 has been isolated and characterized from chickens with IBH [6]. The pathogenicity of this strain has been evaluated in SPF chicken embryos [35] but the pathogenicity of the virus incriminated has never been investigated in chickens.

In the present work, the recently isolated FAdV-11 in Morocco from broiler chicken affected with IBH was selected in order to determine its pathogenicity in specific pathogen free (SPF) chickens by evaluating several parameters including mortality, clinical signs, macroscopic and microscopic lesions and FAdV antibodies response.

## Materials and methods

### Chickens and ethics statement

Eighty 3-day-old SPF chickens were used in this study. All chickens were kept in isolated room in the Biosafety level 3 animal house at the Division of Pharmacy and Veterinary Inputs (NFSA) which is reporting to the National Food Safety Agency in Morocco (NFSAM) and provided food and water *Ad libitum*.

The animal study was undertaken after approval of the NFSAM (i.e the animal welfare authority in Morocco). The study was carried out in strict accordance with the guidance of the NFSAM. After the euthanasia the anatomopathological changes have been documented and presented during post mortem examinations.

### Origin of strain and preparation of inoculum

The FAdV strain used in this study was isolated from liver samples of broiler chickens with IBH in Morocco and it was molecularly identified as belonging to FAdV-D serotype 11(accession numbers MK468898). This isolate was passaged three times in SPF embryonated chicken eggs via chorioallantoic membrane (CAM) route according to standard method [36]. The liver tissues from infected embryos were aseptically collected and homogenized up to 10% (w/v) in 0.01 M phosphate-buffered saline, and clarified by centrifugation at 1900g for 20 min at 4°C. The supernatant was filtered through 0.45μm and 0.2μm filter and mixed with 20 000 units of penicillin and 200μg streptomycin/ml. Titration of the virus was carried out by inoculating serial ten-fold dilutions of liver homogenate into 10-day-old SPF embryonated chicken eggs via the CAM. The median embryo infective dose (EID_50_) of the FAdV isolate was determined using method of Reed and Muench [37]. Liver homogenate with a titer of 10^4^ EID_50_/0.2ml was used as inoculums for the infection.

### Experimental design

Eighty 3-day-old SPF chickens were separated randomly into two groups of 40 birds. One group was inoculated orally with 0.1 ml of inoculums containing 10^4^ EID_50_/0.2ml of virus, the second group was inoculated with 0.1 ml of phosphate-buffered saline (PBS) solution and served as negative controls. Afterwards, the chickens were observed daily for clinical signs. The body weight of all birds was measured at 3, 6, 9, 13, 16, 20, 23 and 28 dpi (day post-infection). At each of this sampling point, five birds from each group were euthanized with intravenous injection of sodium pentobarbital (200 mg/ml) (Dolethal, IPV Morocco) and necropsied. Cloacal swabs were taken from 5 birds per group and conserved in 1ml of antibiotics-phosphate buffered saline solution (1mg /ml streptomycin and 100 000 IU/ ml penicillin). Blood samples were taken from 5 birds per group at same sampling point.

During post mortem examination, livers in particular were investigated for pathological changes and tissues samples of liver, kidney and spleen were collected and stored at– 80°C for virus isolation and DNA detection by PCR while other parts of the same sample tissues were fixed with 10% neutral buffered formalin (NBF) for histopathological examination.

### Histopathology

10%-NBF fixed tissues were embedded in paraffin according to standard methods, and cut into 5μm sections then stained with hematoxylin and eosin, and examined under light microscope for pathological changes.

### Virus isolation

Liver tissues were homogenized in 10% PBS containing 200 U/ml penicillin and 0.2 mg/ml streptomycin. Homogenates were centrifuged at 2000 g for 10 min at 4˚C. The supernatant was filtered through 0.45μm. 500 μl of tissue homogenate was inoculated on confluent chicken embryo fibroblasts (CEF) cells. The inoculum was allowed to adsorb onto the cells at 37˚C for 60 min, after that, 8ml of the maintenance medium containing 2% foetal calf serum (FCS) was added to the culture. The infected flasks and uninfected flaks used as control were incubated at 37˚C under 5% CO2 and the monolayers were observed for cytopathic effect (CPE) daily for 7days. Each sample was passaged up to three times or until cytopathic effect was observed. A sample was considered negative when no cytopathic effect was observed after three passages. The presence of virus in cell culture was confirmed by PCR.

### Viral DNA detection

Viral DNA was extracted from 100μl of tissue homogenate of liver, spleen, and kidney as well as cloacal swabs and infected CEF cell culture by using the Mancherey Nagel Kit (Nucleospin Tissue, Germany). The primers were designed from conserved reported sequences identical to a region of the hexon protein gene of several FAdVs including the L1 region. The amplification was performed as described previously [6]. Positive and negative controls were included with each set of reactions.

### Serology

Blood samples were collected in a 1.5 ml tubes. They were centrifuged at 1900 g for 10 minutes for serum separation. Sera were screened for FAdV antibodies using commercially available ELISA kit (BioChek, UK). Briefly, sera were diluted 1:100 in PBS solution and analysed for presence of IgG antibodies specific for FAdV. Sera with sample/positive (S/P) values above the cut-off level of 0.50 were considered positive according to the manufacturer’s instructions. Each sample was tested twice.

### Statistical analysis

The average body weight and FAdV ELISA titer of orally infected chickens and uninfected chickens at 0, 3, 6, 9, 13, 16, 20, 23 and 28 dpi were recorded and analyzed by Student t test. Statistical differences with P value < 0.05 were considered to be significant.

## Results

### Weight gain

**Fig 1:**
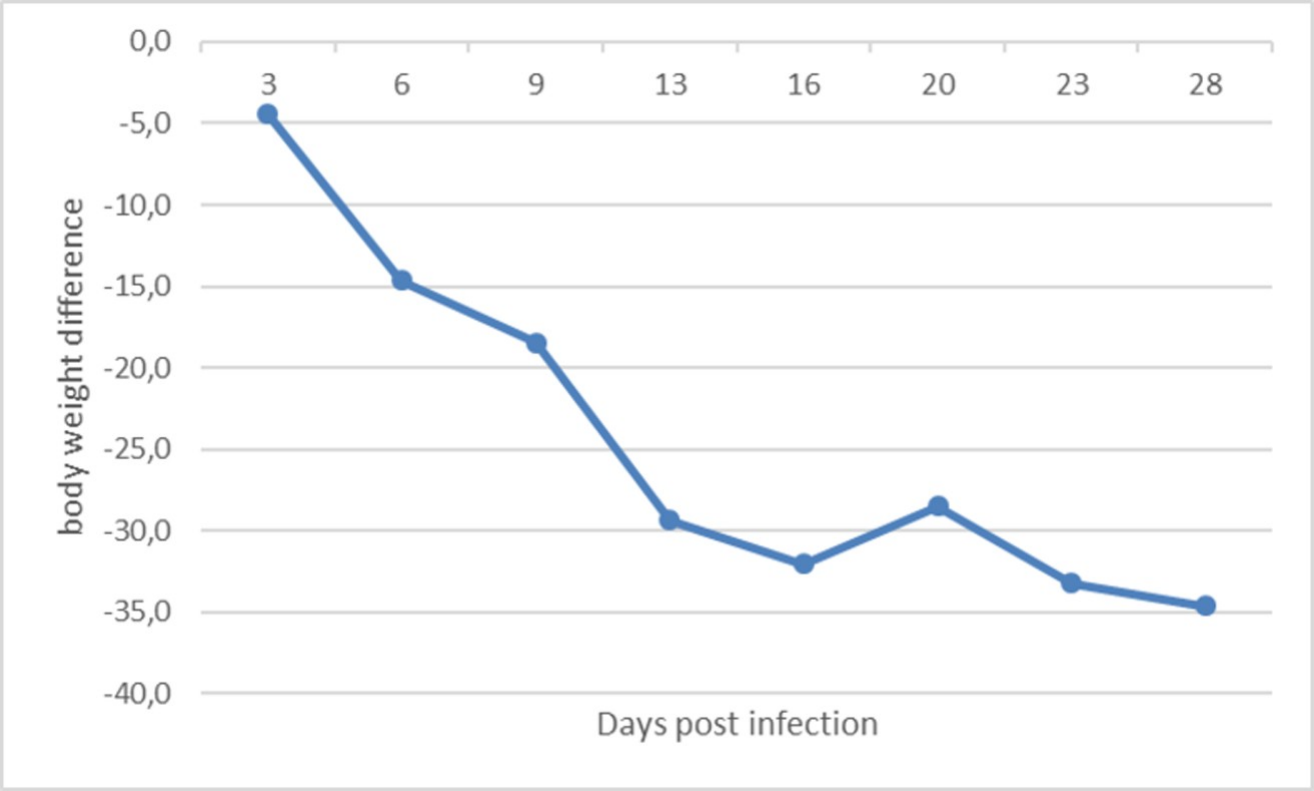
Mean body weights differences from infected SPF chickens to negative controls at 3, 6, 9, 13, 16, 20, 23 and 28 days post infection.

The infected group showed decreased weight gain in comparison to the uninfected control group (Fig 1). The reduction of weight gain was significant from 6 dpi until the end of the experiment (P < 0.05).

### Clinical signs and gross pathology

**Fig 2:**
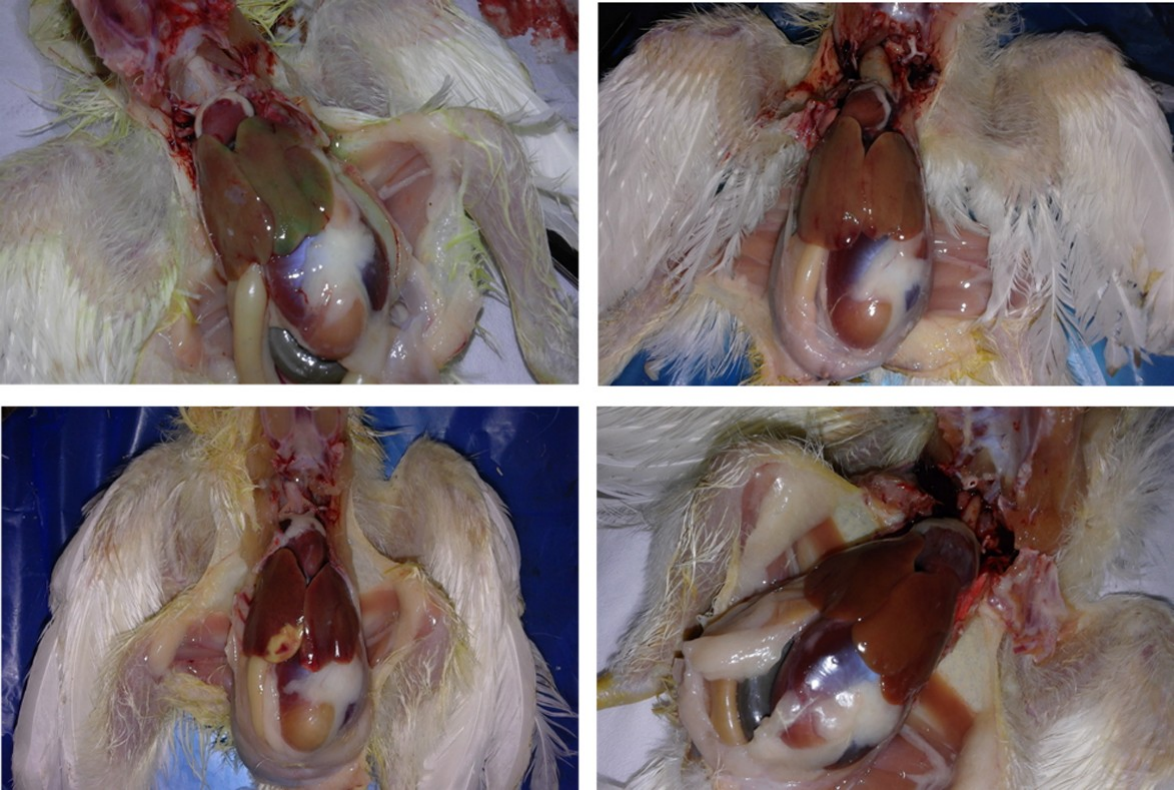
Gross lesions of liver in infected chickens. a: hypertrophied greenish liver of infected chicken at 9 dpi with; b: Pallor and enlargement in liver of infected chicken at 13 dpi with; c: Necrosis lesion in liver at 13 dpi; d: Normal liver of uninfected chicken.

Specific pathogen free chickens infected with FAdV 11(MOR300315 strain) didn’t show any clinical signs during the experiment; only one bird was dead at 4dpi. Chickens in control group did not show any clinical signs or mortality. None of the chickens in infected group showed macroscopic lesions of the liver or other organ at 3dpi. Afterwards, at 6 dpi, more obvious changes were noticed including pallor and enlargement of liver, swelling and slight hemorrhage in kidneys and spleen Table 1. The gross lesions were most severe from 9 dpi to 16 dpi with hypertrophied greenish liver (Fig 2).

**Table 1:** Gross pathological changes in organs collected from infected chickens.

dpi	Hypertrophy and palness of liver	Swollen kidney	Swollen spleen
3	0/5^a^	0/5	0/5
4^b^	1/1	0/1	0/1
6	5/5	5/5	5/5
9	5/5	4/5	4/5
13	5/5	2/5	2/5
16	5/5	0/5	0/5
20	4/5	0/5	0/5
23	5/5	0/5	0/5
28	4/4	0/4	0/4

^a^ No. birds positive/no. birds examined.

^b^One chicken necropsied at 4 dpi was found dead.

### Histopathology

**Fig 3:**
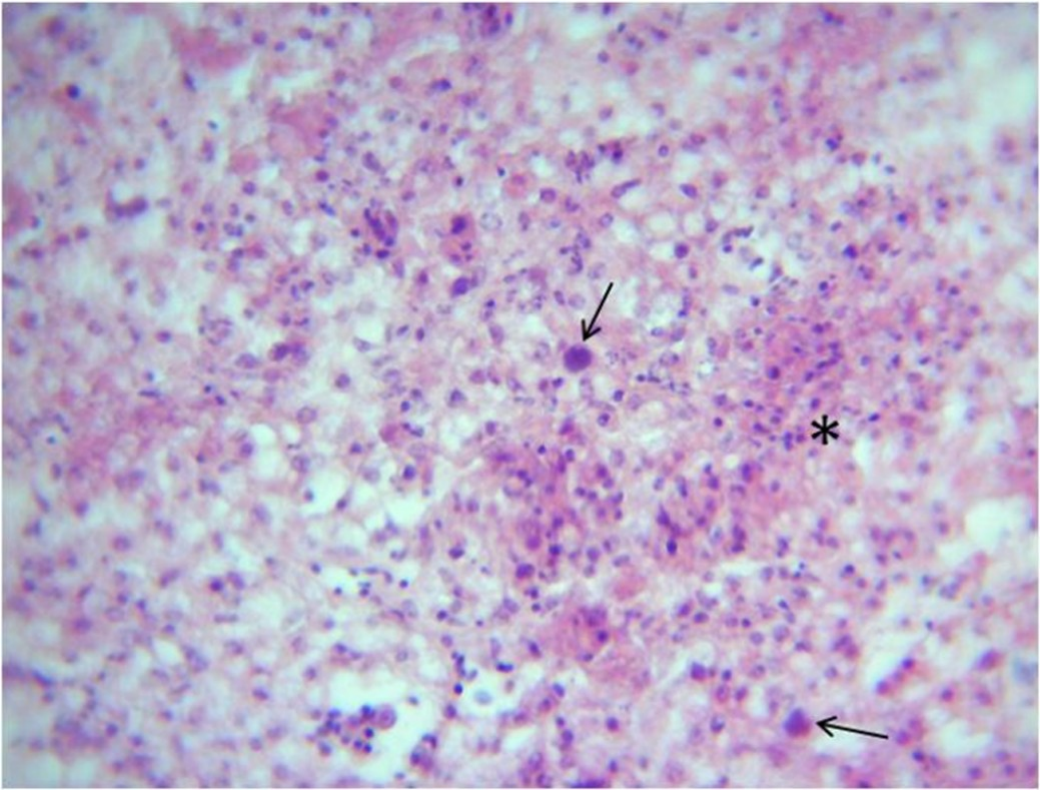
Liver of inoculated chicken at 4dpi–area of severe individual and diffuse hepatic necrosis. Cellular debris necrotic hepatocytes (Asterix) and presence basophilic intra-nuclear inclusion bodies (Arrows). H&E, X400.

**Fig 4:**
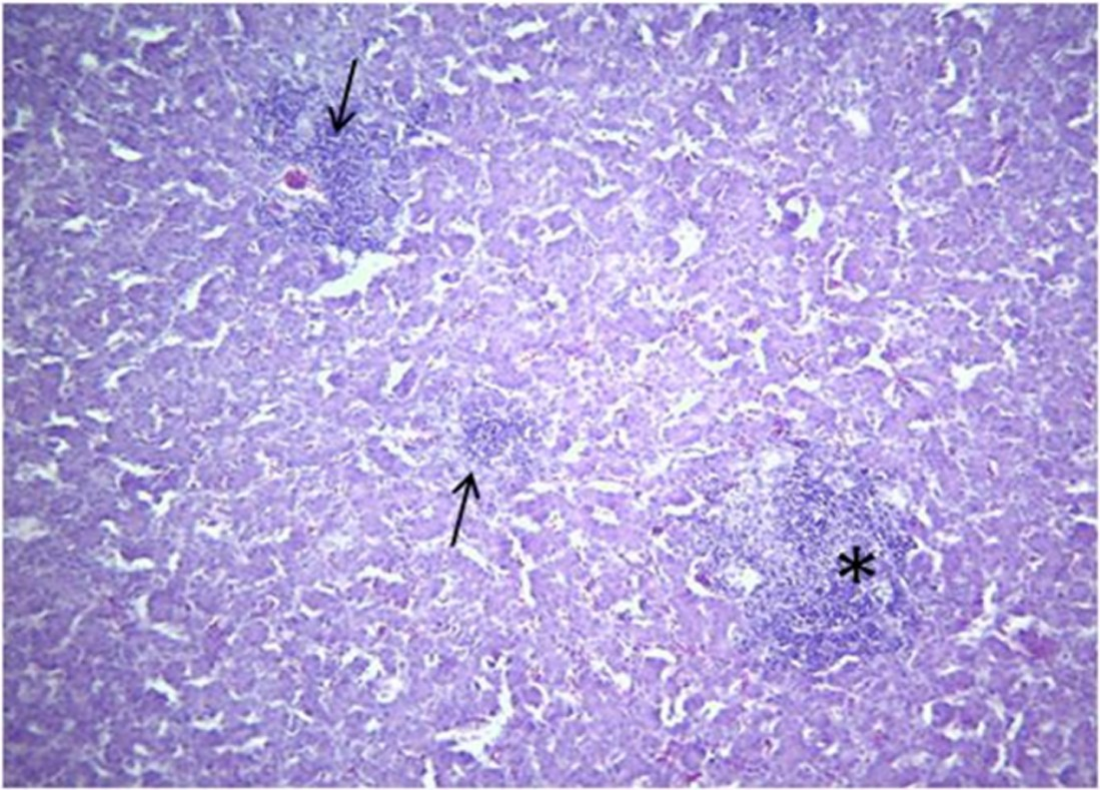
Liver chicken inoculated with FAdV-11 field strain (MOR300315) at 6 dpi -A focus of hepatic necrosis (Asterix) and mixed inflammatory cell infiltrate in the portal spaces (Arrows). H&E, X100.

**Fig 5:**
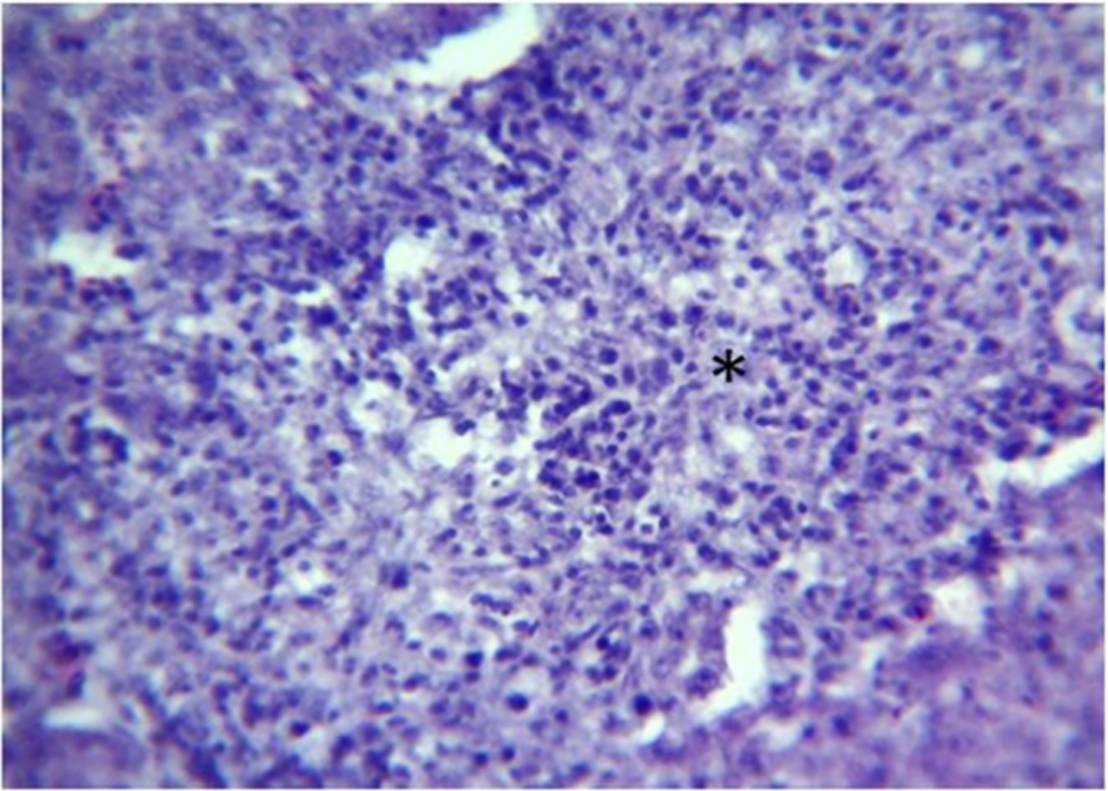
Liver at 6dpi (higher magnification of Fig 4)–foci of hepatic necrosis. Mixed heterophils and lymphocytes with cellular debris of necrotic hepatocytes (Asterix). H&E, X400.

**Fig 6:**
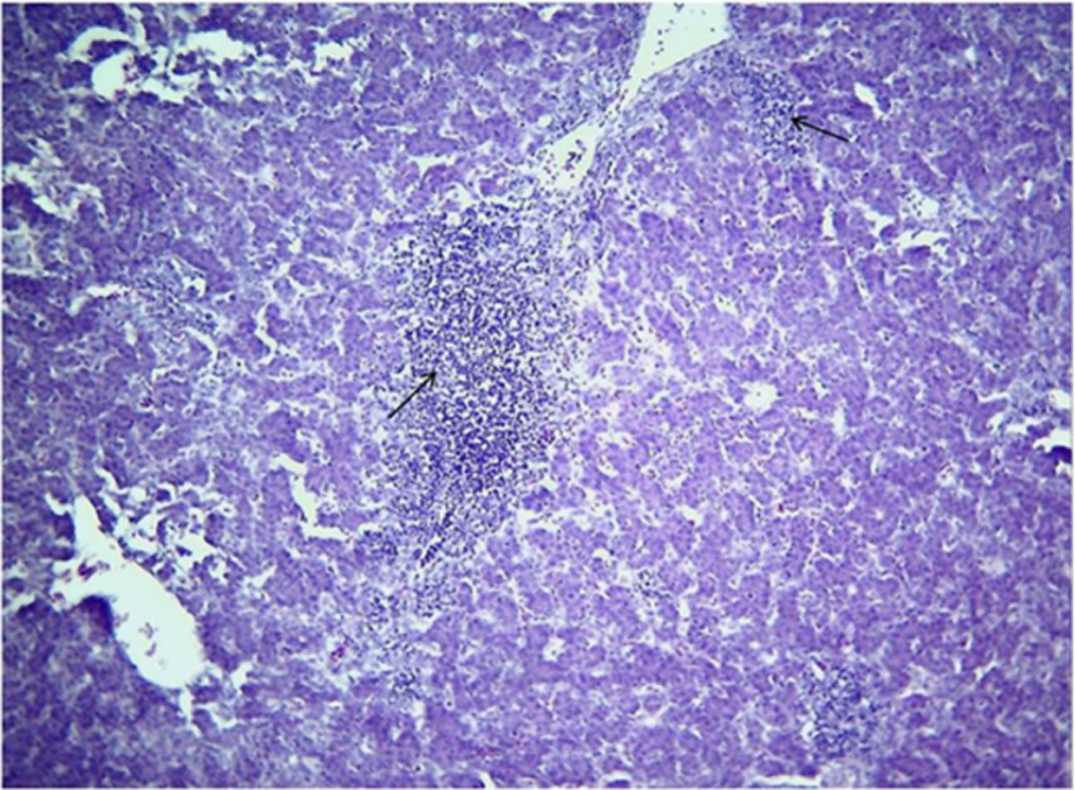
Liver at 9 dpi chick inoculated with FAdV-11 field strain (MOR300315) -Heavy inflammatory cell infiltrate in the portal spaces (Arrows). H&E, X100.

Microscopic changes and level of their occurrence in livers from FAdV-11 inoculated chickens are summarized in Table 2. Liver sample taken from the only one dead chicken at 4 dpi showed severe and extensive hepatic necrosis and basophilic intra-nuclear inclusion bodies within hepatocytes (Fig 3). In liver from chickens necropsied at 6 dpi microscopic changes included multifocal and severe hepatocyte necrosis (5 birds/5) with a variable degree of mixed inflammatory cell infiltrate in the portal spaces mimicking a necrogranuloma appearance (Figs 4–6). No evidence of intra-nuclear inclusion bodies was noted within hepatocytes of examined liver. At 9, 13 and 16 dpi, changes in the liver were restricted to mild to heavy mixed inflammatory cell infiltrate in the portal spaces (Fig 6) and no necrosis of hepatocytes was detected except for 1 bird at 16 dpi. The occurrence of these changes was not observed in chickens after 16 dpi. No microscopic changes were observed in livers from control group chickens as well as in other organs from both inoculated and control group.

**Table 2:** Levels of microscopic change occurrence in livers of chickens inoculated with FAdV (MOR300315 strain) from 4 to 28 days post-challenge.

dpi	Microscopic changes			
	Individual, extensive hepatocyte necrosis	Multifocal hepatocyte necrosis and inflammatory cell infiltrate	Basophilic intranuclear inclusion bodies	Inflammatory cell infiltrate of portal spaces
4^b^	1/1	-	1/1	1/1
6	-	5/5^a^	-	5/5
9	-	-	-	5/5
13	-	-	-	5/5
16	-	1/5	-	3/5
20	-	-	-	-
28	-	-	-	-

^a^ No. birds positive/no. birds examined;

^**b**^Liver sample taken from dead chicken at 4 dpi.

### Virus isolation

CEF cell culture inoculated with homogenates of liver samples collected from infected chicken and dead chicken showed CPE 48 h post-inoculation (hpi). Infected cells were rounding and clumping and started to detach from the monolayers by 72hpi. Whereas no such changes were observed in the uninfected CEFs cell cultures. The presence of the virus in infected cells was confirmed by PCR. Live virus was recovered from 3 to 6 dpi, afterwards the live virus was not detected Table 3.

**Table 3:** FAdV isolation and nucleic acid detection in samples obtained from cloacal swabs and/or organs of infected chickens.

FAdV-11		dpi								
		3	4^b^	6	9	13	16	20	23	28
**Virus isolation from livers**		5/5^a^	1/1	5/5	0/5	0/5	0/5	0/5	0/5	0/4
**DNA detection**	Cloacal swabs	5/5	NE	5/5	0/5	0/5	0/5	0/5	0/5	0/5
	Liver	5/5	1/1	5/5	1/5	0/5	0/5	0/5	0/5	0/5
	Kidney	3/5	1/1	3/5	1/5	1/5	1/5	0/5	0/5	0/5
	Spleen	3/5	1/1	3/5	1/5	1/5	0/5	0/5	0/5	0/5

Samples from orally infected chickens with FAdV-11 were investigated at 3, 6, 9, 13, 16, 20, 23 and 28 days post infection (dpi) by conventional PCR.

^a^No. of positive samples / no. of tested samples.

^b^Organ samples taken from a dead chicken at 4 dpi.

NE not examined.

### PCR

Viral DNA in sampled tissues was detected by PCR and the results were summarized in Table 3. FAdV was detected from cloacal swabs, liver, kidney and spleen at 3 dpi. Virus excretion in internal organ was noticed until 13 dpi. Whereas all tissues samples collected from dead chicken at 4dpi were positive for viral DNA.

### Serology

#### Antibody response

**Fig 7:**
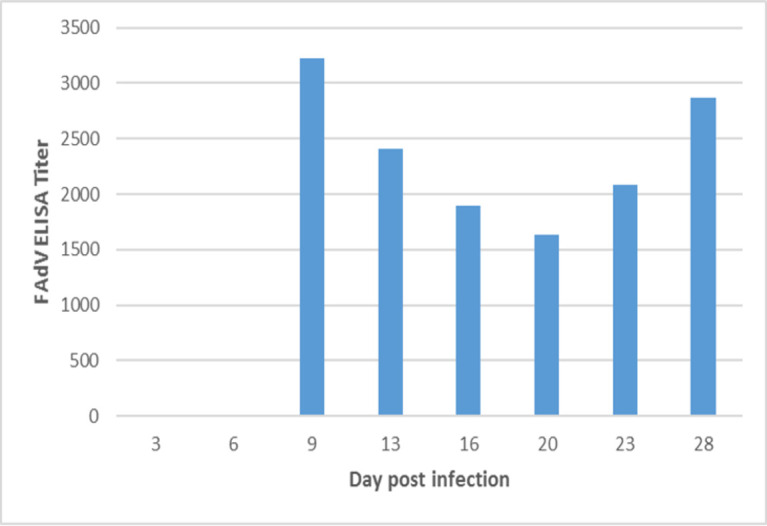
Antibody response to viral proteins in chickens inoculated with FAdV isolate MOR300315 by an oral route as measured by ELISA. Five sera samples from inoculated chickens were randomly collected at 3, 6, 9, 13, 16, 20, 23 and 28dpi. The bar representing the titer at 3 and 6 dpi are not visible as antibodies were not detected.

Serum antibodies against the inoculated strain were not detected at 3 and 6 dpi. Antibodies response to viral proteins appeared at 9, 13, 16, 20, 23 and 28 dpi with average titer of 3223, 2403, 1897, 1633, 2083 and 2863, respectively (Fig 7). FAdV-specific Abs were not detected in any birds of the control group throughout the experiment.

## Discussion

In recent years, increasing clinical cases of FAdV infections have been concerned and many FAdV strains related to IBH have been identified in many countries [11, 15, 25, 33, 38–43]. Some of them were highly pathogenic to chickens and were associated to considerable economic losses [15, 26, 33, 44].

In Morocco, outbreaks of IBH were notified in broiler and layer chickens since 2013, causing serious economic impact on the poultry industry [2–6]. FAdV-11 strains were isolated and characterized from field IBH cases [6]. The pathogenicity of this strain was evaluated in SPF chicken embryos and found highly pathogenic [35]. However, the pathogenicity of the isolated viruses has never been evaluated in chickens.

In the present study, FAdV-11 strain isolated from affected chickens in Morocco was used to examine its pathogenicity and tissue tropism following experimental infection of SPF chickens through a natural route. This is the first report of pathogenicity of FAdV-11 strain circulating in Morocco in chickens.

We showed that FAdV-11 produced gross and histological lesions in the absence of any immunosuppression using oral inoculation of 3-day-old SPF chickens. Very few experimental studies have been able to successfully induce IBH using natural routes of infection in young SPF chickens [8].

The lack of clinical sign, even with the extent of the lesions seen in inoculated chickens with FAdV-11 is in concordance with natural occurring outbreaks of IBH during which a few clinical signs are observed [8, 45, 46]. Similarly, no obvious clinical signs were shown by SPF chickens after their experimental inoculation by FAdV-11 [26, 33, 47].

The decreased weight gain and gross lesions observed in livers of birds seen in FAdV-11(MOR300315) inoculated SPF chickens were comparable to those obtained in other experiments [28, 44] as well as in natural infections with FAdV-11 [5, 6, 33, 42, 48–50]. Poor weight gain was mainly attributed to malabsorption [42, 48, 49].

Histologically, the main changes found in livers of chickens inoculated with FAdV-11 included hepatic necrosis associated with the presence of basophilic intra-nuclear inclusion bodies within hepatocytes, and mixed inflammatory cell infiltrate in the portal spaces. Hepatic necrosis was extensive and severe as early as 4 dpi (in 1 naturally dead chick) and was associated with the presence of basophilic intra-nuclear inclusion bodies within hepatocytes. These changes are highly characteristic of both natural and experimental infection with FAdV-11 [6, 26, 28, 33, 42, 50]. At 6 dpi, birds showed rather a multiple foci of necrosis with mixed inflammatory infiltrate resembling to necrogranulomas, however no basophilic intra-nuclear inclusion bodies within hepatocytes could be detected at this stage. Despite the absence of this characteristic feature of IBH, FAdV could be recovered from all birds by PCR and virus isolation on CEF indicating a strong link between necrotic foci and the inoculated virus. Analogously, it was reported that in the absence of the characteristic inclusions in liver tissues from FAdV inoculated chickens, the presence of multifocal hepatic necrogranulomas could be used as an indicator of successful FAdV isolation, because it correlated 100% with PCR results [51]. In the present work, significant and characteristic changes of IBH could be reproduced in chickens at 4–16 dpi, thus highlighting the pathogenic potential of FAdV-Moroccan isolate to experimentally induce changes in the liver which is the primary target organ.

In other organs such as kidneys and spleen, no lesions could be detected in inoculated birds at any time post-challenge. Nevertheless, in natural FAdV infection, a tubulo-interstitial nephritis characterized by mononuclear infiltrate of the renal interstitial tissue along with tubular necrosis with or without the presence of basophilic intra-nuclear inclusion bodies within tubular cells has been well documented [2].

The FAdV was reisolated in chicken embryo fibroblast (CEF) cell culture inoculated with liver tissue homogenates prepared from collected liver samples of experimentally infected chickens at 4 and 6 dpi. The cytopathic effect (CPE) observed is similar to that induced in CEF cell cultures inoculated with different strains of FAdV [6, 52–54]. This finding confirms the replication of FAdV-11 in infected tissue.

In parallel, viral DNA was detected by PCR in liver, kidney and spleen from 3 dpi until 13 dpi. Indeed, similar trend has been previously reported in other FAdV-11 experimental infection studies in chickens [26, 28, 33, 47]. In addition, a correlation between macroscopic lesions, histological finding and viral DNA detection in the liver of FAdV-11 challenged chickens was found at 6 and 9 dpi. The most extensive microscopic lesions in liver and high number of positives by PCR in that target organ were detected at 6 dpi which is in accordance with the results of earlier investigations [26, 28, 33].

The detection of viral DNA in cloacal swabs at 3 and 6 dpi confirm the excretion of the virus in the fomities very early during the experiment and no later than 6 dpi. In other trials, the viral shedding of FAdV-11 could be detected until 15 dpi [33]. Moreover, it has been shown that the duration of virus shedding in the faeces do not correlate with the pathogenicity of the virus, but with viral dose administred [33, 44, 55, 56].

The antibodies response against FAdV isolate (MOR300315) was detected from 9 to 28 dpi with significant differences between inoculated chickens and negative control (P<0.05). This result is in concordance with other experiments reported by earlier workers where the FAdV antibodies response after inoculation of SPF chickens with FAdV-11 strain, appeared at 14 and 21 dpi [15]. In other experimental infection of chickens with FAdV-8, the serum antibodies were present from 7 dpi [57].

In conclusion, this is the first study demonstrating that the FAdV-11 isolated from outbreaks of IBH in Morocco, is pathogenic in chickens, without any predisposing pathogens or immunosuppressive factors, such as poor feed or environmental conditions. This finding has confirmed also the possibility of horizontal transmission occurring in the first few days of life. In addition, this study has provided a comprehensive description of the chronology of development of lesions after experimental infection with FAdV-11 and its relationship with the presence of the virus. These results are the starting point for further investigations into the pathogenicity of FAdVs to provide useful information for development of the efficient vaccine against IBH in Morocco.

## Supporting information

S1 File Body weights of infected SPF chickens and negative controls at 3, 6, 9, 13, 16, 20, 23 and 28 days post infection. (PDF)
