# Supplementary material for: Pathogenicity of field strain of fowl aviadenovirus serotype 11 isolated from chickens with inclusion body hepatitis in Morocco
Source: PLoS One. 2021 Dec 16;16(12):e0261284. doi: 10.1371/journal.pone.0261284 (PMC8675708; doi:10.1371/journal.pone.0261284)

Body weights of infected SPF chickens and negative controls at 3, 6, 9, 13, 16, 20, 23 and 28 days post infection

Control	Infected	Control	Infected	Control	Infected	Control	Infected	Control	Infected	Control	Infected	Control	Infected	Control	Infected
3 dpi		6 dpi		9 dpi		13 dpi		16 dpi		20 dpi		23 dpi		28 dpi	
78	60	84	78	98	98	136	108	170	132	214	192	243	206	314	275
60	66	90	70	110	78	160	124	158	134	214	196	252	204	314	295
70	62	102	70	118	104	136	114	182	120	224	195	252	214	316	283
74	62	78	76	102	80	138	116	158	152	190	193	242	218	312	262
64	62	80	83	108	78	140	124	180	138	214	172	252	219	314	282
66	60	92	64	103	102	152	123	170	118	218	170	252	221		
74	68	84	72	115	92	160	122	192	162	220	186	242	215		
59	62	82	64	116	92	152	114	182	158	190	185	252	226		
66	56	74	72	95	92	138	112	192	152	214	160	252	218		
62	50	94	64	98	88	160	118	180	158	218	190	248	214		
60	64	78	72	98	80	152	124	170	162	222	192				
78	74	84	78	110	82	140	124	192	160	190	190				
62	60	90	79	118	100	136	124	170	156	214	178				
70	60	102	78	102	82	138	100	158	158	217	190				
74	60	78	74	108	94	140	110	180	144	221	164				
64	56	80	78	103	86	136	123	182	150						
66	70	92	72	115	84	160	106	192	152						
74	50	84	72	116	92	140	124	182	134						
61	52	82	60	98	94	138	120	158	126						
66	58	74	60	95	92	152	106	180	122						
62	60	94	75	98	90	152	124								
60	70	78	76	108	68	140	98								
78	70	84	66	110	72	136	92								
60	50	90	76	102	106	138	124								
70	62	102	56	118	102	160	124								
74	72	78	66	103	78										
64	66	80	60	115	80										
66	66	92	74	98	74										
74	62	84	78	95	88										
60	52	82	66	116	86										
68	60	74	64												
63	58	94	66												
60	68	78	76												
64	52	90	70												
66	64	80	70												

	74	66														
	60	62														
	66	72														
	62	70														
	61	70														
**Mean body weight**	**66,5**	**62,1**	**85,3**	**70,7**	**106,3**	**87,8**	**145,2**	**115,9**	**176,4**	**144,4**	**212,0**	**183,5**	**248,7**	**215,5**	**314,0**	**279,4**
**Body weight difference**	**-4,4**		**-14,5**		**-18,5**		**-29,3**		**-32,0**		**-28,5**		**-33,2**		**-34,6**	

**Day post infection**	3	6	9	13	16	20	23	28
**Body weight difference**	-4,4	-14,5	-18,5	-29,3	-32,0	-28,5	-33,2	-34,6

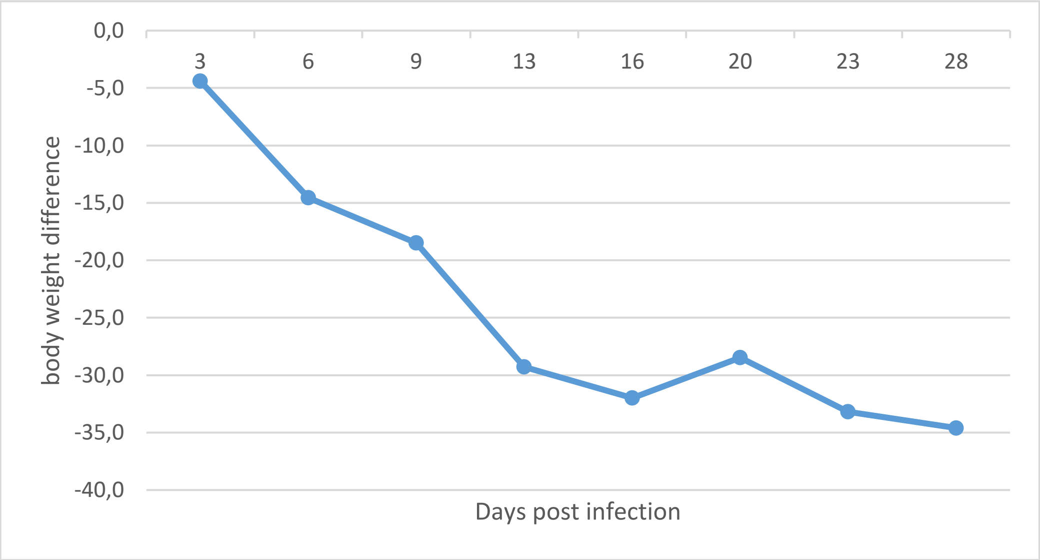

Supplement: S1 File — (PDF) [file pone.0261284.s001.pdf]
